# Mirror-like brain responses to observed touch and personality dimensions

**DOI:** 10.3389/fnhum.2013.00227

**Published:** 2013-05-29

**Authors:** Michael Schaefer, Michael Rotte, Hans-Jochen Heinze, Claudia Denke

**Affiliations:** ^1^Department of Neurology, Otto-von-Guericke University MagdeburgMagdeburg, Germany; ^2^Department of Anesthesiology and Intensive Care Medicine, Charité – Universitätsmedizin BerlinBerlin, Germany

**Keywords:** somatosensory cortex, personality, touch, NEO-FFI, mirror network, fMRI

## Abstract

The last years have shown a growing interest in research on the neural mechanisms for perceiving and understanding social interactions. Only very recently, a role for somatosensation in social perception has been suggested. Numerous studies reported vicarious responses in the primary somatosensory cortex (SI) and other areas merely when seeing others being touched. Moreover, it has been demonstrated that these vicarious somatosensory responses can be linked with inter-individual differences in empathy. However, beyond empathy other personality traits have been shown to interact with social perception and behavior. Here we tested if personality traits according to the Five-Factor-Model interact with vicarious activation in somatosensory brain regions. We conducted a functional magnetic resonance imaging (fMRI) study in which subjects viewed video clips showing simple non-painful touch to a hand and a control condition including the same visual and motion parts. Results revealed vicarious somatosensory activation when viewing the touched hand, as expected. Vicarious activation in SI showed a trend for a positive correlation with the personality trait openness to experience. Moreover, mirror-like responses in the insula were strongly correlated with the personality trait conscientiousness, suggesting links to processes of self-control. We conclude that vicarious brain responses to seen touch seem to interact with personality traits.

## Introduction

In the last years numerous studies tried to reveal the neural mechanisms for perceiving and understanding social interactions (Cacioppo and Decety, 2011). Understanding of the conspecific's experiences is crucial for social behavior. According to the mirror neuron theory this understanding is accomplished by an internal simulation of other's experiences we are observing (Rizzolatti et al., 2001). Recent studies revealed mirror-like responses not only for actions, but also for touch. Thus, it has been shown that merely viewing touch involves the observers' somatosensory cortices. For example, Bufalari et al. (2007) reported that somatosensory evoked potentials (SEPs) were modulated by the observation of a touched hand. They found increased P45 amplitudes during pain observation (a needle penetrating a hand) and decreased P45 amplitudes during touch observation. Studies employing fMRI, magnetoencephalography, or transcranialmagnetic stimulation (TMS) support the results of vicarious somatosensory activation when observing touch (Keysers et al., 2004; Blakemore et al., 2005; Ebisch et al., 2008, 2011; Gazzola and Keysers, 2008; Schaefer et al., 2009; Pihko et al., 2010; Wood et al., 2010; Bolognini et al., 2012; Meyer et al., 2011; Kaplan and Meyer, 2012).

It has been argued that we perceive the social world differentially according to our personality traits. Consequently, recent studies suggest that mirror-like responses are linked with personality traits. For example, Fecteau et al. (2008) reported a relationship between mirror responses in the motor system and psychopathic personality traits. Avenanti et al. (2009) employed TMS to demonstrate that somatomotor responses to others' pain were influenced by the observers' empathy traits. In addition, several studies suggest that mirror-like responses in somatosensory brain regions are prone to interindividual differences. Osborn and Derbyshire (2010) report that when observing clips or pictures of injuries about one-third of participants experience feeling pain on the corresponding part of their own body, while the remaining two-thirds report negative feelings without a sense of somatic pain. A subsequent fMRI experiment revealed vicarious activity in SI and secondary somatosensory cortex (SII) associated with the images of injuries, but only in those participants who experienced localized vicarious pain.

Moreover, recent studies discuss an association for SI and empathy beyond the observation of painful stimulation. Ruby and Decety (2004) reported that empathy and perspective taking in complex social events are associated with activation in SI. Hooker et al. (2010) presented social scenes in an fMRI experiment and showed a correlation of somatosensory areas on the left postcentral gyrus with empathy. Gazzola et al. (2006) reported that a group of more empathic subjects compared with a group with lower empathy scores activated the mirror system (including the somatosensory cortices) more strongly. Our previous study supported these results by showing that mirror-like responses in SI during observation of simple nonpainful touch are linked with empathy (Schaefer et al., 2012a).

The above-mentioned studies refer to interindividual differences in vicarious somatosensory engagement only with regard to empathy. This makes sense in particular in studies examining somatosensory responses when witnessing painful stimulation. But recent studies showed interindividual differences in empathy even for participants observing stimuli not related to pain (e.g., Gazzola et al., 2006; Schaefer et al., 2012a). Based on these results one could hypothesize that vicarious somatosensory activations may also be affected by more general personality traits. This is supported by a recent study showing that personality may depend on primary somatosensory cortex activity. Using neuromagnetic source localization, this study demonstrated that the personality dimension extraversion predicted the strength of somatosensory brain responses when receiving nonpainful touch (Schaefer et al., 2012b). The results support an earlier study reporting a relationship of extraversion with SI activity (Shagass and Schwartz, 1965). The relationship of the personality trait extraversion with primary somatosensory cortex activity can be explained by neurobiological assumptions of personality (e.g., Eysenck, 1967; DeYoung et al., 2010). Based on these results we here wanted to examine if somatosensory cortex activity elicited by merely observed touch is similarly prone to interindividual differences in extraversion. Since extraversion is related to the perception of social stimuli and the mirror neuron system is discussed as a neurobiological foundation of social perception, we hypothesized that interindividual differences in extraversion may also influence mirror-like responses in the brain. Thus, we tested if responses in somatosensory brain regions when seeing someone else being touched are affected by personality traits according to the Five-Factor-Model (FFM). The FFM is a factor-analytic approach describing the human personality in five core dimensions, which are extraversion, neuroticism, agreeableness, conscientiousness, and openness to experience. Extraversion is displayed by a tendency to experience positive emotions and includes a high degree of sociability, assertiveness, and talkativeness. Neuroticism is linked to the tendency to experience negative emotions, involving anxiety, self-consciousness, and irritability. Agreeableness is linked to altruism, including traits such as cooperation, compassion, and politeness. Conscientiousness is reflected by being disciplined, organized, and achievement-oriented. Openness to experience involves active imagination, aesthetic sensitivity, attentiveness to inner feelings, preference for variety, and intellectual curiosity (Costa and McCrae, 1992).

In order to test our hypothesis we reanalyzed data from our previous fMRI study (Schaefer et al., 2012a), in which we presented video clips showing a hand receiving tactile stimulation with a paintbrush and as a control condition the same picture and motion parts, but without seeing the hand being stimulated (analogue to Keysers et al., 2004; Schaefer et al., 2009). We hypothesized that the vicarious activation of somatosensory brain regions during the observation of touch is linked with interindividual differences according to the FFM. Given the results of recent studies showing relationships of empathy with SI (Ruby and Decety, 2004; Gazzola et al., 2006; Hooker et al., 2010; Osborn and Derbyshire, 2010; Schaefer et al., 2012a), we expected an interaction of personality especially with vicarious activity in SI. More in detail, we assumed a relationship of extraversion with mirror-like responses in SI, because activity in SI has been linked with extraversion (Shagass and Schwartz, 1965; Schaefer et al., 2012b). Thus, we argue that the simulation of touch is similarly affected by the extraversion dimension as actual real touch. Based on previous results (Schaefer et al., 2012b) we hypothesized that more introverted participants should show stronger mirror-responses in SI.

Beyond mirror like responses in SI or SII, insula activation during observation of touch has been reported (Blakemore et al., 2005; Morrisson et al., 2011; Schaefer et al., 2012a). In addition, based on experiments investigating affective responses, numerous studies showed interindividual differences in insula activation (Mazzola et al., 2010; Guiliani et al., 2011; Banissy et al., 2012; Bauer et al., 2012). Furthermore, studies investigating the relationship between conscientiousness and learning suggest a link for this personality trait to self-related cognitions (e.g., Martocchio and Judge, 1997; Lee and Klein, 2002). For example, Martocchio and Judge (1997) suggested a model of two mediating constructs, self-deception and self-efficacy, which are hypothesized to mediate the relationship between conscientiousness and learning. Their findings indicated that conscientiousness was positively related to self-efficacy as well as to self-deception, whereas self-efficacy was positively and self-deception negatively linked to learning. However, both psychological constructs were linked to conscientiousness. Since these concepts can be described as self-related cognitions and the insula is known to represent self-awareness (Craig, 2009), sense of agency (Farrer and Frith, 2002) and sense of body ownership (Tsakiris et al., 2006), we hypothesized relationships of the insula with self-related personality dimensions (in particular, conscientiousness), whereas more social aspects of personality dimensions (extraversion, agreeableness) should not be related to insula activation when seeing someone else being touched.

## Materials and methods

### Participants

Seventeen out of the 22 participants that participated in the previous study (Schaefer et al., 2012a) were included in the current analyses. Two were discarded due to technical problems; one further participant was excluded due to poor data quality in the empathy questionnaire. In addition, the present study was unable to collect NEO-Five-Factor Inventory data from two further participants, resulting in a final N of 17 participants (nine females, mean age 26 years, range 23–39 years). All participants were right-handed native German volunteers with no neurological or psychiatric history. The study adhered to the Declaration of Helsinki and was approved by the local human subjects committee. Informed written consent was obtained from all subjects.

### Procedure

The stimuli consisted out of video clips depicting a right hand (egocentric viewpoint) and a moving paintbrush. There was one experimental condition (= touch observation condition), one control condition, and one additional condition to localize somatosensory brain regions (= real touch condition). The video clips (and the real touch condition) lasted for 18 s and were followed by resting periods of 15 s ± 3 s.

**Figure 1:**
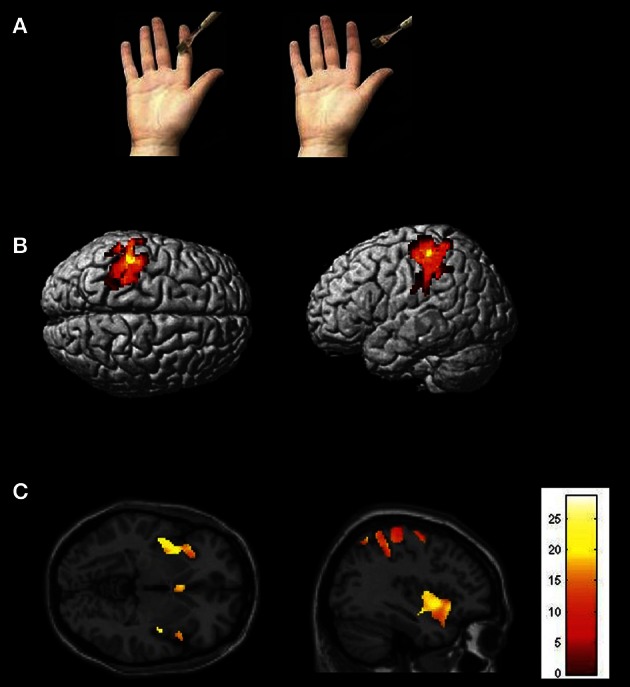
**(A)** Conditions and types of stimuli used in the experiment. The picture on the left depicts the experimental condition (touch to a hand); the picture on the right shows the control condition (the paintbrush does not hit the depicted hand). **(B)** Statistical map showing common brain activation in left SI for receiving real touch (>resting baseline) and observing touch (>control) (random-effects analysis, *p* < 0.05, FWE corrected). **(C)** Statistical map demonstrating activation in insula (and other brain areas, masked with real touch > baseline). Areas of significant fMRI signal change are shown as color overlays on the T1-MNI reference brain.

In the touch observation condition video clips showed a hand repeatedly being touched on the index finger by a paintbrush. In the control condition the paintbrush made identical motions as in the touch observation condition except that in the former, the brush stroked on the side of the index finger, but did not touch the hand (see Figure 1). In all conditions, a right hand was stimulated. The same visual stimuli and motion frequency (1/s) were applied in all video clips. The motion of the paintbrush was vertical in about 90 percent of all trials and horizontal in about 10 percent. Participants were required to press a key with their left hand to report the number of vertical strokes at the end of each video clip (analogue to Schaefer et al., 2009). Two fingers were used to indicate the number of vertical strokes. The key was custom-made and had two buttons. Participants were instructed to answer as soon as they saw the asterisk marking the beginning of the resting block. Yes and no buttons were randomized over the trials. The task was designed to ensure that subjects paid attention to the videos (analogue to Blakemore et al., 2005; Schaefer et al., 2009).

Visual images were back-projected to a screen at the end of the scanner bed close to the subject's feet. Subjects viewed the images through a mirror mounted on the birdcage of the receiving coil.

In the real touch condition the participant's right hand was repeatedly touched by a paintbrush during the fMRI scan. Subjects were not able to watch the stimulation. The manner and frequency of brushing were identical to that shown in the touch observation videos. Participants were instructed to focus a fixation asterisk.

Each experiment consisted out of three runs. Each run included nine experimental and nine control blocks. In addition, three real touch blocks for localizing somatosensory brain areas were added to each run. Video stimuli and real touch stimulation were presented in a random order and were counterbalanced over the runs. The experiment lasted for about 45 min.

After the experiment, participants were asked to complete a German version of the NEO Five-Factor Inventory (NEO-FFI, Borkenau and Ostendorf, 1993). Furthermore, subjects completed a German version of the Interpersonal Reactivity Index (IRI, Davis, 1983), which is a 28-item self-report survey consisting out of four subscales: Empathic Concern (EC), Personal Distress (PD), Perspective Taking (PT), and Fantasy (F). EC describe a person's tendency to have feelings of sympathy and concern for others. PD measures the tendency to which someone feels a negative emotion. PT assesses the extent to which someone cognitively imagines a situation from the other person's point of view. The F subscale describes the tendency to project oneself into the place of fictional characters in books and movies. Results regarding the empathy measures were published in our previous study (Schaefer et al., 2012a).

### fMRI data acquisition and analysis

The functional imaging was conducted by using a 1.5 T scanner (General Electrics Signa LX, Fairfield, Conneticut, USA) to conduct functional imaging (gradient echo T2-weighted echo-planar images; *TR* = 2 s, *TE* = 35 ms, flip angle = 80 degrees, FOV = 20 mm). Functional volumes consisted of 23 slices. Each volume comprised 5 mm slices (1 mm gap, in plane voxel size 3.125 × 3.125 mm). For anatomical reference a high-resolution T1-weighted structural image was collected (3D-SPGR, *TR* = 24 ms, *TE* = 8 ms).

Functional imaging used the technique of Statistical Parametric Mapping Software (SPM5, Wellcome Department of Imaging Neuroscience, University College London, London, UK). Prior to statistical analysis, the images were corrected for subject motion, spatially normalized to a standard anatomical space with a resampled voxel size of 3 mm (MNI, Montreal Neurological Institute template), and then spatially smoothed with a Gaussian kernel of 6 mm full-width half maximum.

Statistical parametric maps were calculated using multiple regression with the hemodynamic response function modeled in SPM5. We examined data on the individual subject level by using a fixed effects model (the three runs were concatenated for each subject). For each subject we calculated the contrast (blockwise) observing touch relative to control (*t*-test). The resulting parameter estimates for each regressor at each voxel were then entered into a second-level analysis. Functional analyses were based on the contrasts (*t*-tests) between observation of touch and the control condition, using random-effects models. To investigate common activations between real touch and the mere observation of tactile stimulation, the contrasts (observation of touch relative to control) were inclusively masked by the contrast of real touch minus resting baseline (at *p* < 0.05).

We report regions that survived correction for multiple comparisons over the whole brain [family-wise error (FWE) correction at *p* < 0.05]. We used the SPM Anatomy toolbox for anatomical interpretation of the functional imaging results (Eickhoff et al., 2005).

Scores of the personality traits were tested for possible correlations (Pearson) with the parameter estimates for voxels in the somatosensory region of interest (maximum peak in left SI for contrast touch observation relative to control condition, masked with real touch relative to resting baseline). Furthermore, we tested possible correlations with personality traits for left SII, left and right insula, and left premotor region (maximum peaks for touch observation relative to control, masked with real touch). Results of the correlation data were corrected for multiple tests (Bonferroni). Thus, considering five regions of interest and nine different scales (IRI and NEO-FFI), correlations with *p* < 0.001 were described as significant.

Behavioral responses were analyzed by comparing the task accuracy (stroke count) between experimental and control conditions (*t*-test). Task accuracy was defined as number of video clips in which participants correctly identified the number of vertical strokes. Furthermore, we tested task accuracy with personality dimensions (IRI and NEO-FFI) for significant correlations. The results were Bonferroni corrected for nine scales (IRI and NEO-FFI), thus, results with *p* < 0.005 were considered as significant.

Finally, we tested the behavioral responses (task accuracy) with BOLD signal changes in SI and insula (all correlations Pearson) (Bonferroni correction for two scales, *p* < 0.025).

## Results

### NEO-FFI results

The mean value for extraversion was 29 ± 6 (mean ± standard deviation; range 15–36); for neuroticism 19 ± 9 (range 6–32); for openness to experience 32 ± 6 (range 21–41), for agreeableness 34 ± 5 (range 28–38) and for conscientiousness 33 ± 7 (range 17–46). There was a negative correlation between extraversion and neuroticism (*r* = −0.74, *p* < 0.05).

### Behavioral results

The overall accuracy of the task performance during fMRI scanning was 80% (standard deviation ±15%; across all conditions; mean for experimental condition: 79 ± 15%; mean for control condition: 81 ± 14%). There were no significant differences in subjects' performance (i.e., accuracy of stroke count) over the experimental conditions [touch observation, control condition: *t*_(16)_ = −0.46, *p* = 0.65]. Accuracy of the behavioral responses was not associated with personality dimensions (all *p* > 0.10). In addition, reaction times were not correlated with personality measures (all *p* > 0.10). None of our participants stated to have imagined the seen hand as the own hand.

### Imaging results

Analysis of the fMRI data showed that the contrast real touch relative to resting baseline yielded in activation of contralateral postcentral gyrus (SI), bilateral parietal operculum (SII/parietal ventral area), the precentral gyrus (BA4/6), the insula, the lateral temporo-occipatal cortex, the superior parietal /intraparietal cortex, and thalamus (*p* < 0.05, FWE corrected).

Brain regions overlapping with observed touch (touch observation > control, masked with real touch > resting baseline) showed significant activation in postcentral gyrus (SI/BA 2), SII, premotor cortex (BA44, BA6), SMA, ventral anterior (Deen et al., 2010) or mid (Taylor et al., 2009) insula, superior parietal lobe, superior temporal gyrus, and cerebellum (see Figure 1 and Table 1).

**Table 1:** **Results of random effects analysis (at *p* < 0.05, FWE corrected; L, left hemisphere; R, right hemisphere; masked with real touch > baseline) for contrast touch observation relative to control**.

**Contrast**	**Brain region**	**MNI coordinates**			**Peak *t*-value**
Touch observation > control	L SI	−38,	−36,	52	16.46
	L premotor cortex/BA44	−56,	8,	12	15.85
	L premotor cortex (BA6)	−28,	−10,	60	20.36
	L precentral gyrus (BA6)	−54,	4,	38	11.34
	R SMA (BA6)	6,	14,	60	15.61
	L SMA (BA6)	−4,	4,	46	23.69
	L insula	−40,	2,	−4	23.01
	R insula	44,	12,	−6	16.48
	R SII/sup. temp. gyrus	58,	−32,	22	12.90
	L SII	−54,	−30,	2	9.45
	R sup. parietal lobe (BA7A)	22,	−60,	64	15.05
	L sup. parietal lobe (BA7A)	−32,	−60,	60	18.72
	L sup. temp. gyrus	−62,	−42,	22	12.56
	Cerebellum	−8,	−44,	−31	22.80

The contrast control relative to touch observation failed to show any significant voxels.

**Figure 2:**
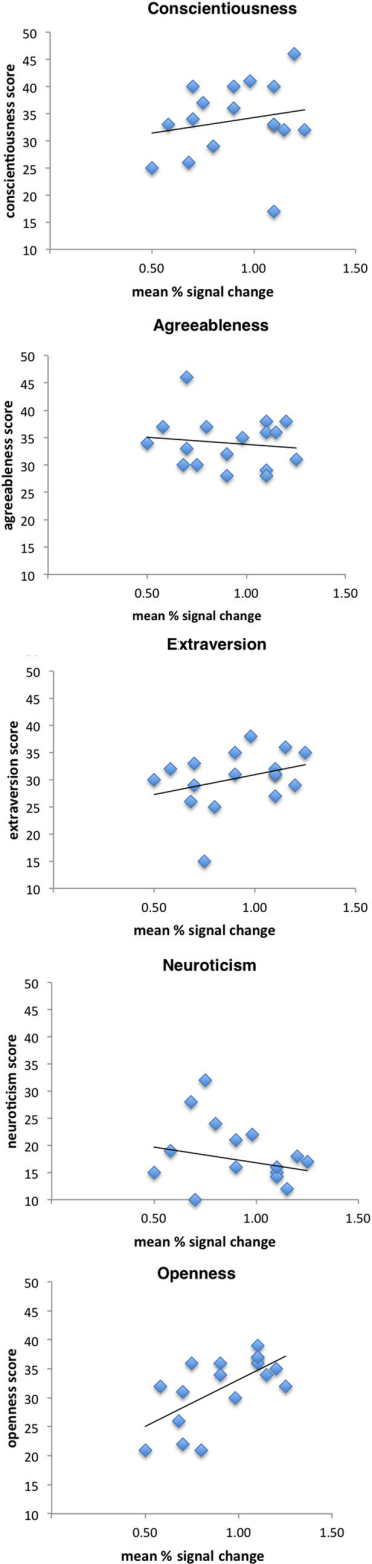
**Correlation scatterplots for personality dimensions openness to experience, agreeableness, extraversion, neuroticism, and conscientiousness of the FFM and left SI activation when observing a touched hand (see text for further details)**.

Figure 2 shows scatterplots of brain responses (parameter estimates) in left SI with NEO-FFI scores of the five factors. We used the parameter estimates for the maximum activation (peak voxel) of the cluster in left SI, which has been assigned to BA2 (Eickhoff et al., 2005). Activity in SI correlated with openness to experience with a trend for significance (*r* = 0.64, *p* = 0.006), but not with any other personality measure (neuroticism: *r* = −0.20, *p* = 0.44; agreeableness: *r* = −0.13, *p* = 0.62; conscientiousness: *r* = 0.19, *p* = 0.47; extraversion: *r* = 0.32, *p* = 0.21).

**Figure 3:**
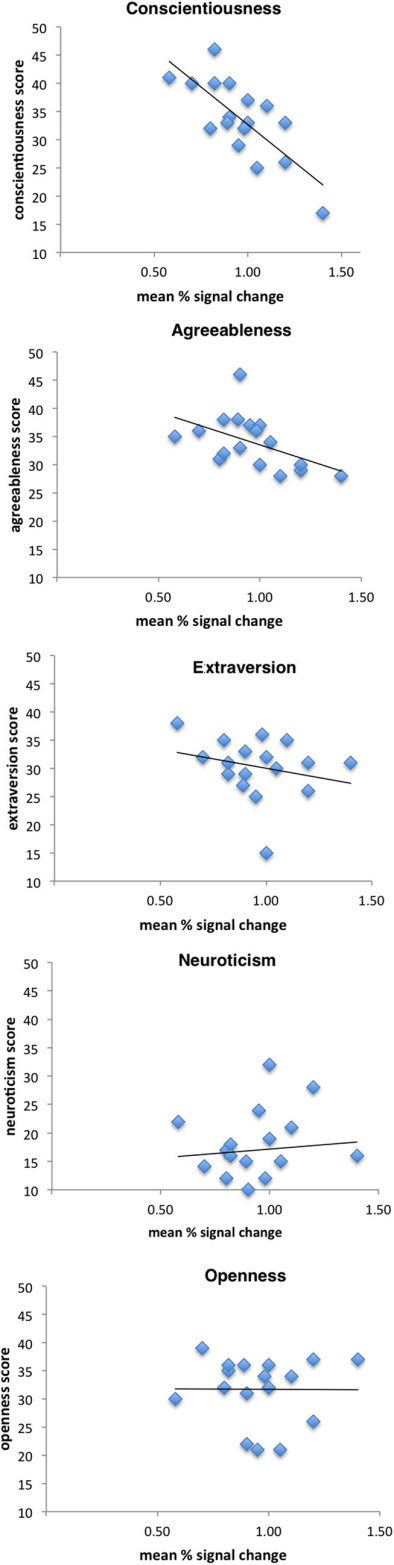
**Correlation scatterplots for personality dimensions and left mid insula activation when observing a touched hand.** Results demonstrated a significant negative correlation with the personality dimension conscientiousness (see text for further details).

Figure 3 depicts scatterplots of vicarious brain responses for observed touch in left anterior/mid insula (peak activation) and NEO-FFI scores of the five factors. Results revealed that activity in insula was strongly significantly correlated (negatively) with the personality factor conscientiousness (*r* = −0.76, *p* < 0.001). No other personality dimension revealed significant correlations with insula activation (neuroticism: *r* = −0.06, *p* = 0.83.; agreeableness: *r* = −0.50, *p* = 0.04; openness: *r* = −0.01, *p* = 0.98; extraversion: *r* = −0.25, *p* = 0.33).

Vicarious responses in SII, right insula or in premotor cortex failed to show any significant relationships with personality dimensions.

Furthermore, we tested if the five personality dimensions were related to the empathy subscales of the IRI. The empathy subscale PT, which has been shown to be linked with vicarious activation in SI in our previous study (Schaefer et al., 2012a), was correlated positively with openness to experience (*r* = 0.38), but failed to reach the level of significance (*p* = 0.14). Colinearity statistics revealed VIF (variance inflation factor) values of 1.1 for PT and openness. Since these values are relatively low, it seems unlikely that multicollinearity effects may have affected the correlation coefficient reported above. Further correlations revealed no significant results (PT with neuroticism: *r* = 0.16, with extraversion: *r* = 0.06, with agreeableness: *r* = −0.44, with conscientiousness: *r* = 0.09; PD with neuroticism: *r* = 0.34, with extraversion: *r* = −0.28, with openness: *r* = −0.42, with agreeableness: *r* = −0.05, with conscientiousness: *r* = −0.04; EC with neuroticism: *r* = 0.34, with extraversion: *r* = −0.50, with openness: *r* = 0.03, with agreeableness: *r* = −0.20, with conscientiousness: *r* = 0.19; F with neuroticism: *r* = −0.009, with extraversion: *r* = 0.008, with openness: *r* = 0.44, with agreeableness: *r* = −0.06, with conscientiousness: *r* = 0.31).

Furthermore, correlations between task performance and vicarious somatosensory activation in SI (and insula activation, respectively) revealed no significant correlations (analogue data analysis to the correlation analysis with personality dimensions).

We also correlated NEO-FFI personality dimensions with SI activation resulting from real touch. Results revealed a trend for a significant relationship with openness (*r* = 0.61, *p* = 0.009). Other personality dimensions were not linked to SI activity (extraversion: *r* = −0.26, *p* = 0.31; neuroticism: *r* = 0.30, *p* = 0.23; agreeableness: *r* = −0.31, *p* = 0.61; conscientiousness: *r* = −0.11, *p* = 0.66; PD: *r* = −0.45, *p* = 0.08; EC: *r* = −0.00, *p* = 0.98; F: *r* = 0.43, *p* = 0.10; PT: *r* = 0.30, *p* = 0.26).

## Discussion

Recent studies reported mirror-like responses in the somatosensory cortices when subjects witness the sensations, actions and somatic pain of others. Remarkably, it has been reported that these vicarious activations in SI are affected by interindividual differences in empathy (e.g., Schaefer et al., 2012a). The current study aimed to test if mirror-like responses in somatosensory brain regions are linked to personality dimensions beyond empathy. Results revealed no significant correlations of SI activity and personality dimensions, but a trend for significance for openness to experience. Mirror-like responses in insula were significantly (negatively) correlated with the personality trait conscientiousness.

Based on previous studies linking activity in SI with extraversion (Shagass and Schwartz, 1965; Schaefer et al., 2012b), we hypothesized that mirror-like responses in SI may similarly be associated with this personality dimension. Our results did not support this hypothesis. Moreover, SI activation for real touch expressed a negative correlation with extraversion, as expected, but failed to reach the level of significance. One explanation for this lack of significant relationship with real touch might be that we stimulated the right hand. Our previous study demonstrated significant correlations for SI with extraversion when touching the left hand. Touch to the right hand revealed a similar negative correlation, but this relationship was weaker and failed to reach the level of significance. The previous study explained this effect with a special role for the right hemisphere in processing social information. Furthermore, since neuromagnetic source imaging and BOLD responses do not measure exactly the same neurophysiological processes, they may not be fully comparable with respect to the activation level we report (dipole moments vs. signal change in BOLD response). In addition, different kinds of stimulation (pneumatically vs. paintbrush) on different sites of the hand were used. In general, correlational analysis of BOLD activity with behavioral responses should be done carefully, since behavioral tests often require many more participants than fMRI experiments usually provide (the same argument applies for correlations with neuromagnetic data). This seems to be in particular true for correlations with personality measurements. In order to address this question we here used conservative corrections for multiple tests (Bonferroni).

Is the lack of correlation between extraversion and seen touch driven by the lack of a significant correlation of extraversion with touch alone? We think that this is not likely because in contrast to touch alone (and our previous study), which expressed a negative correlation, the relationship for merely observed touch was positive. Thus, it seems that vicarious somatosensory responses in SI may be unaffected by the personality factor extraversion. Future studies are needed to examine if the observation of more complex social interactions may be linked to this dimension or if mirror-like responses in SI are independent of this personality factor. In addition, it should be tested if the observation of touch on a left hand would affect the relationship with the personality dimensions. Future research may also include further control conditions, for example, touch to animated relative to unanimated objects, which could refer more specifically to the social domain.

While our hypothesis of a correlation with extraversion was not confirmed, we found a positive correlation with a trend for significance between mirror-like responses in SI and openness to experience. Interestingly, SI activation during the real touch condition revealed a trend for a positive correlation with openness, too. However, both correlations failed to reach the level of significance. Thus, these results remain tentative and speculative. Future studies are needed to reveal if these trends point to meaningful relationships.

Why may openness to experience be related to vicarious touch? We speculate that both the correlations for observed as well as for real touch might be caused by attention effects, which is in accordance with the description of the openness personality trait (DeYoung et al., 2005). The reason why our previous study (Schaefer et al., 2012b) did not find any relationship with openness might be the different stimulation technique. While our previous study used an automatic pneumatic stimulation device, the current experiment used touch from a paintbrush moved by an experimenter. Recent results showed that the response in SI can be modified by affective information on the experimenter (Gazzola et al., 2012). Hence, the stimulation paradigm in the current study may have resulted in stronger attention to the stimulation, which seems to have driven the correlation with the personality dimension openness.

Previous studies already demonstrated that vicarious responses in somatomotor brain areas were affected by empathy (e.g., Schaefer et al., 2012a). So how is empathy related with the FFM? Several studies found interrelations between both the FFM and dispositional empathy. For example, Mooradian et al. (2011) report interrelations of the four empathy subscales of the IRI (Davis, 1983) with the FFM. The empathy subscale EC was closely related to agreeableness and PD closely linked to neuroticism. Perspective taking correlated with all five domains in the NEO-FFI, pointing to interstitial relationships to the five factors. Our results failed to show a significant correlation of the empathy subscale perspective taking (or of any other empathy subscales) with openness to experience, making it unlikely that empathy (perspective taking) rather than openness may have caused the correlation between SI and openness. Why were there no relationships between IRI and NEO-FFI in our study while other report correlations? Studies such as Mooradian et al. (2011) report results from a much bigger sample than our study, while our sample size may be typical for imaging studies. However, the low VIF values in our study make it unlikely that multicollinearity effects may have affected the correlation between openness and SI activity.

The current study reports mirror-like responses also for somatosensory brain regions beyond SI. Insula activation was closely associated (negatively) with the personality trait conscientiousness. Thus, the less the participant scored on the dimension conscientiousness, the more the insula was engaged while observing the touched hand. What is the role of the insula in our experiment? Since the insula is closely connected with ascending internal body signals, recent studies have proposed a role of the insula for the sense of self. For example, Modinos et al. (2009) let participants reflect upon their own personal qualities as compared to those of an acquaintance. Results revealed activation in left anterior insula uniquely associated with self-reflection. Karnath et al. (2005) suggested that the (postular) insular cortex is integral to self-awareness, in particular coding information on the subject's feeling of being vs. not being involved in a movement (similar Farrer and Frith, 2002). Thus, mirror-like responses in insula in our study seem to be linked to processes of self-awareness or -reflection. In order to differentiate between self and other a sense of self has to be maintained when mirroring (or simulating) seen touch. This seems to be warranted by the insula.

But how is the personality dimension conscientiousness related to this function? Conscientiousness has been described to reflect the tendency to inhibit impulses in order to follow rules. It is opposed to impulsivity and distractibility (Costa and McCrae, 1992; DeYoung et al., 2010). Thus, participants characterized by high impulsivity and distractibility seem to require strong insula activation in order to preserve a sense of self while observing the touched body part. In contrast, participants scoring high on conscientiousness are less impulsive or distractible. Consequently, those subjects may demand only little insula activation in this mirror experiment.

So far, only few studies examined neural correlates for conscientiousness. DeYoung et al. (2010) employed data from structural MRIs and linked conscientiousness with activity in the (lateral) prefrontal cortex, which has been related to the ability to plan and voluntary control of behavior. The authors explain this result with the association of conscientiousness with effective self-regulation at multiple levels of complexity. The results of the present study extend these results by demonstrating that conscientiousness also seems to be linked with functions of self-regulation in the insula during the simulation of observed touch to an alien body. We speculate that this interaction may be grounded on improved connections in the mirror network, on top-down processes (attention), or on both (Gazzola et al., 2006).

While we here argue for links between personality and vicarious somatosensory brain responses, alternative explanations for our results should also be taken into account. For example, one could argue that openness or conscientiousness may generally increase (or decrease, respectively) the cortical activation level. Nevertheless, since openness correlated only with activity in SI, not with any other clusters activated by the sight of touch, it seems unlikely that the association between openness and SI might be explained by a general increase of cortical activity. Similarly, conscientiousness corresponded only with insula activation (and this relationship was negative). Furthermore, task effects might explain our results. For example, participants scoring high on openness to experience simply may pay more attention to the task, resulting in stronger somatosensory responses. This objection might be supported by the fact that higher attention is one of the crucial features in people scoring high on openness. The objection of a possible link to task performance may be even stronger for subjects scoring high on conscientiousness, a personality trait that is known to be related to the ability to follow rules. However, we found no relationship of openness to experience or conscientiousness with the performance of the task. In addition, task performance was independent of BOLD activation in SI and insula. Furthermore, conscientiousness was negatively related with BOLD responses. Thus, it seems unlikely that task performance may have caused the relationship between personality and somatosensory response. In addition, the real touch conditions might have influenced the experimental and control conditions. This seems unlikely since we used relatively long blocks and resting periods. Furthermore, experimental and control conditions included a task, while the real touch condition was passive. However, we used the real touch condition only for localizing somatosensory brain areas. Last, motor responses due to button presses or effects of motor planning may have influenced our results. Thus, activity related to the planning and execution of the button press could have flown into the video conditions or the baseline. This activity could have created noise, which might even be related to personality measures in a systematic way. However, we believe that an influence is unlikely since participants used the left hand for button presses, while the video hand was a right hand. Furthermore, motor related activity should have affected both the touch as well as the non-touch conditions. Finally, response times were not correlated with personality measures.

Despite SI and insula, other brain regions known to be involved by viewing touch events did not show any relationships with personality traits. For example, SII and premotor region showed no significant relationship with personality. The lack of a correlation with vicarious premotor activity may be explained by the minimal motor content in our experimental paradigm (instruction to count the strokes of the paintbrush).

The present study examines relationships between mirror-like responses to observed touch and personality traits. However, based on the present data we feel unable to explain the direction of these correlations. Thus, it remains unclear if vicarious brain responses “cause” the parameter values of the personality traits or if the personality traits “produce” higher mirror-like responses. Future studies are needed to address these questions.

### Conflict of interest statement

The authors declare that the research was conducted in the absence of any commercial or financial relationships that could be construed as a potential conflict of interest.
